# Factors associated with pre-marital sexual debut among unmarried high school female students in bahir Dar town, Ethiopia: cross- sectional study

**DOI:** 10.1186/1742-4755-11-40

**Published:** 2014-05-31

**Authors:** Yeshalem Mulugeta, Yemane Berhane

**Affiliations:** 1 Department of Public Health, College of Medicine and health Sciences, Bahir Dar University, P.O. Box 79, Bahir Dar, Ethiopia; 2 Addis Continental Institute of Public Health, Addis Ababa, Ethiopia

**Keywords:** Sexual debut, Youth, Unmarried female students

## Abstract

**Background:**

Pre-marital sexual debut increase the risk of sexually transmitted infections (STIs) including HIV/AIDS and unwanted pregnancy. It may also affect their school performance and completion rate. In spite of this fact, number of unmarried female students who started sexual debut is increasing from time to time. However, information on the extent of pre-marital sexual debut and associated factors were not well studied and documented in the study area where pre-marital sexual debut is largely condemned. Therefore this study was conducted to assess the magnitude and associated factors of pre-marital sexual debut.

**Methods:**

School based cross-sectional survey was conducted from May 10-13/2012. A total of 1123 unmarried high school female students were selected by multi- stage sampling technique. Data were collected using structured, self administered questionnaire. Descriptive statistics, binary and multivariable logistic regression analyses were used to identify factors associated with pre-marital sexual debut.

**Results:**

Among unmarried high school female students 30.8% reported pre-marital sexual debut. The major associated factors were frequent watching of pornographic video [AOR = 10.15, 95% CI: (6.63, 15.53)], peer pressure [AOR = 2.98, 95% CI: (1.57, 5.67)] and chewing khat [AOR = 8.99, 95% CI: (3.84, 21.06)].

**Conclusion:**

Significant proportion of unmarried high school female students have started pre-marital sexual debut. The finding suggests the need for communicating and supporting school students to help them make informed and safer decisions on their sexual behavior. Therefore, Bahir dar city administration health and education bureau should design persistent and effective health education to decrease pre-marital sexual debut in unmarried female students.

## Introduction

In Ethiopia, about 16% of unmarried female youth reported sexual debut [1]. Of which 24.6% had two or more sexual partners but only 10% of them used condom during sexual intercourse [1]. In Amhara region, where the study was conducted, pre-marital sexual debut was reported as early as 12 to 13 years [2,3]. It is not uncommon for female students to establish sexual networks with local businessmen and uniformed men in return for money and gifts [2,3].

Bahir dar town has a wide range of high-risk groups including Female sex workers, University students, high school students and migrant workers. Cross-generational and transactional sex between young girls and older men is the common practice in the town. High school girls also have sex with older men in return for gifts and money [4].

Female youth who have begin pre-marital sexual debut earlier appear more likely to have sex with high risk partners or multiple partners and are less likely to use condom and other contraceptives [5]. Therefore, they are vulnerable to sexually transmitted infections including HIV/AIDS and unwanted pregnancy [6,7].

Unwanted pregnancy may be associated with greater likelihood of early motherhood, unsafe abortion, and other pregnancy related complications. These events in turn, increase risk of morbidity and mortality both in the mother and the child. Furthermore, early motherhood tends to impede female students academic performance, and eventually dropout from school. It often results in reducing their economic opportunity [8].

Different studies identified inconsistent factors of pre-marital sexual debut. These factors include pear pressure, Living arrangement, age of students, religiosity, school performance, having pocket money, substance use, watching pornographic video, place of family residence and parental educational status [3,9-24].

Although HIV/AIDS affects all segments of the population, young people were largely affected by this disease and young females were largely affected than young males [25]. In Ethiopia according to antenatal care sentinel surveillance (ANC), the prevalence of HIV/AIDS among young people of age 15–24 years was 2.6%; specially Bahir dar town had the highest reported HIV prevalence rate [26].

Pre-marital sexual debut has the above serious immediate and long term consequences to female students in the contexts where the practice is widely condemned. Furthermore, there is double standard cultural expectations of virginity at marriage for females while lesser cultural pressure on males. However studies on magnitude of pre-marital sexual debut and its associated factors are scarce. Thus, this study was conducted with the objective of assessing the extent of pre-marital sexual debut and the factors associated with it among unmarried high female school students.

## Methods

School based cross-sectional survey was conducted in Bahir Dar town from May 10-13/2012. The town is the capital city of Amhara National Regional State, located 564 km northwest of Addis Ababa (capital city of Ethiopia). In Ethiopia, high school includes education from the ninth to the twelfth grade. In Bahir dar town there were three high schools with a total of 3984 students during the time of the study. Of which 1845 were females and 1773 were unmarried females.

The required sample size of the study was calculated using single population proportion formula by considering the following assumption: a 95% confidence level, 5% marginal error, 44% proportion of pre-marital sexual debut [27], consider high non response rate because the topic is sensitive issue (18% non response rate) and 2.5 design effect. The final sample size was 1123 students.

Study participants were unmarried female students selected from the three high schools. Students were selected using multi stage sampling technique and probability proportionate to size of the students from each school. List of sections from each school used as a sampling frame. Sample sections were selected randomly using simple random sampling technique. Students from each section were selected again using lottery method from list of students in each selected section. The usual age range of students enrolled in Ethiopia high school is 15 to 24 years, however, in some instances; students could be younger or older than this age range. For this study, students who were not within this age range were ineligible and did not participate in the study.

Data were collected by adapted [28], pretested structured facilitator guided self administered questionnaire. The questionnaire was developed in English then translated in to Amharic (local language) then back to English to check consistency. Data were collected from all students selected from one school simultaneously in one day to overcome information contamination. Data collection facilitators and supervisors were recruited and trained. They helped in seating students in the classrooms designated for completing the survey and provided explanation on the study procedures and tools. They also ensured complete privacy during completing the questionnaire.

The dependent variable was pre-marital sexual début; it refers first penetrating heterosexual intercourse. While the independent variables were socio-demographic characteristics of respondents, school related and parental factors. Peer pressure in this study is presence of peer who started pre-marital sexual debut and encourage inexperienced female students to experiment it.

Data were cleaned, coded and entered on to EPi-Info version 3.5.2 Statistical package soft ware. Data were exported to SPSS version 16 program for analysis. Proportion of reported pre-marital sexual debut was computed. Logistic regression was used to control confounding effects. P value ≤ 0.2 was taken as a cut-off point for selecting variables for the logistic regression model. Residence, age, watching pornographic video, frequency of watching pornographic video, peer pressure, frequency of attained religious service, average mark, chewing khat, respondents currently living arrangement, pocket money, and religion were included in the final adjusted model. The model was built with backward elimination to avoid multicollinearity. P-values less than 0.05 was considered statistical significant. Education about disadvantage of pre-marital sex was given. Incentive in terms of money was not given for the participants.

The study was conducted after securing ethical approval of Institutional Review Committee of Gondar University. Written permission to conduct the study in the selected schools was obtained from the Regional Education Bureau. An informed consent was secured from each participant. The confidentiality was maintained throughout the study by excluding personal identifiers from the data collection form.

## Results

A total of 1123 students participated in the study. Of which 1093 filled the questionnaire fairly completed, making the response rate 97.3 percent. The mean age of respondents was 18.17 ± 0.93 years. The majority of participants were Orthodox Christian followers, 938 (85.8%) and from Amhara ethnic group, 1017 (93.0%); had some amount of pocket money, 881 (80.6%); and had parents from urban areas, 916 (83.8%) (Table 1).

**Table 1:** Socio-demographic characteristics of unmarried high school female students in Bahir Dar town, from May 10-13/2012

**Variable**	**Frequency (n = 1093)**	**Percentage (%)**
**Age**		
16-18 years	751	68.7
19-20 years	330	30.2
21-24 years	12	1.1
**Religion**		
Orthodox	938	85.8
Muslim	100	9.1
Protestant	46	4.2
Catholic	9	0.8
**Attending religious service**		
Occasionally	375	34.3
3 or more times a week	356	32.6
Daily	304	27.8
Not at all	58	5.3
**Ethnicity**		
Amhara	1017	93.0
Tigray	30	2.7
Agew	22	2.0
Oromo	18	1.6
Guragie	6	0.5
**Pocket money**		
Had pocket money	881	80.6
Had no pocket money	212	19.4
**Respondents currently live with**		
Both parents	604	55.3
Mother	132	12.1
Alone	118	10.8
Relatives	83	7.6
Garden	58	5.3
Friends	57	5.2
Father	41	3.8
**Parental place of residence**		
Urban	916	83.8
Rural	177	16.2
**Average mark**		
60-79%	757	69.3
80-89%	177	16.2
50-59%	100	9.1
90-100%	57	5.4

About 337 (30.8%) of unmarried female students reported having sexual debut. The mean age of sexual debut was 16.46 ± 1.43 years. The minimum age of sexual debut was 13 years and maximum age of sexual debut was 24 years.

Among students who started pre-marital sexual debut; nearly half, 159 (47.2%) had more than one sexual partner and two third, 216 (64.3%) of them use condom during sexual intercourse. Of which only one third, 71 (32.9%) reported consistent use of condom. One fourth, 82 (24.4%) of students who started pre-marital sexual debut had pregnancy. From those who were pregnant, 73 (89%) of them had history of abortion and 9 (11%) of them gave birth (Table 2).

**Table 2:** Pre-marital sexual debut of unmarried high school female students in Bahir Dar town, from May 10-13/2012

**Variable**	**Frequency**	**Percentage (%)**
**Start pre-marital sexual debut (n = 1093)**		
No	757	69.2
Yes	337	30.8
**Number of sexual partner (n = 337)**		
One	178	52.8
Two	94	27.9
Three or more	65	19.3
**Use of condom (n = 337)**		
Yes	216	64.1
No	121	35.9
**Frequency of condom use (n = 216)**		
Always	71	32.9
Occasionally	145	67.1
**History of pregnancy (n = 337)**		
No	255	75.7
Yes	82	24.3
**History of abortion (n = 82)**		
Yes	73	89.0
No (give birth)	9	11.0

**Table 3:** Factors associated with pre-marital sexual debut among high school female students in Bahir Dar town, from May 10-13/2012

**Factor**	**Premarital sex**		**COR (95% C/I)**	**AOR (95% C/I)**
	**Yes (%)**	**No (%)**		
**Age**				
16- 18 years	193(25.7)	558(74.3)	1.00	1.00
19-24 years	144(42.1)	198(57.9)	2.10(1.60,2.75)	2.13(1.40,3.25)
**Frequency of watching pornographic video**				
≥2 times a week	268(65.4)	142(34.6)	9.66(4.71,19.82)	10.15(6.63,15.53)
≤ one times a week	69(10.1)	614(89.9)	1.00	1.00
**Peer pressure**				
Yes	249(58.3)	178(41.7)	9.19(6.84,12.35)	2.98(1.57,5.67)
No	88(13.2)	578(86.8)	1.00	1.00
**Students chewing khat**				
Yes	84(89.4)	10(10.6)	24.7(12.66,48.45)	8.99(3.84,21.06)
No	253(25.3)	746(74.7)	1.00	
**Frequency of attained religious service**				
≥ 2 times a week	120(18.2)	540(81.8)	0.22(0.16,0.29)	0.50(0.28,0.89)
Occasionally	217(50.1)	216(49.9)	1.00	1.00
**Average mark**				
50-59	61(61)	39 (39)	8.68(3.84,19.64)	8.12(1.67,39.52)
60-79	221(29.6)	536(70.8)	2.29(1.10,4.73)	3.53(0.90,13.87)
80-89	46(26)	131(74)	1.95(.89,4.27)	5.25(1.14,24.09)
90-100	9(15.3)	50(84.7)	1.00	1.00

AOR =Adjusted Odds Ratio; COR=Crude Odds Ratio; 95% CI=ninety five percent confidence interval.

The logistic regression analysis showed that pre-marital sexual debut was more likely to occur among students chewing khat [AOR = 8.99, 95% CI: (3.84, 21.06)], watching pornographic video [AOR = 10.15, 95% CI: (6.63, 15.53)] and those had peer started pre-marital sexual debut [AOR = 2.98, 95% CI: (1.57, 5.67)]. Students those attained religious services two or more times a week were five times less likely to practice pre-marital sexual debut than those attained the service less than two times a week [AOR = 0.50, 95% CI: (0.28, 0.89)] (Table 3).

## Discussion

Pre-marital sexual debut predispose students to sexually transmitted infections including HIV and unwanted pregnancy [29]. In spite of this fact, 30.8% of unmarried high school female students have initiated pre-marital sexual debut. This finding was similar with a study conducted in Gondar preparatory school [30]. On the other hand, It was relatively higher than studies done in high school students of Addis Ababa, Jimma, Eastern Ethiopia and Rural South Africa [11,17,31,32]. This difference might be due to the time gap between the studies, advancement of technology that enhance watching pornographic video and substance use (khat, alcohol, cigarette] which might increase the probability to be engaged in pre-marital sexual activities [33].

Age of respondents was associated with pre-marital sexual debut; those students whose age >18 years were 2 times more likely to start pre-marital sex compared to students age <18 years [AOR = 2.13, 95% CI: (1.40, 3.25)]. This finding is in agreement with study conducted in Rural South Africa, China, Jamaica and India [9,11,17,34]. The age difference for pre-marital sexual debut in this study might be older (>18 years) youth might have stay sexually mature for a long period of time. Exposure to sexual risk factors for a prolonged period would be encourage to start pre-marital sexual debut [35].

Watching pornographic video is a risk factor for pre-marital sexual debut [33]. In this study, frequency of watching pornographic video was associated with pre-marital sexual debut; students who watch pornographic video two or more times a week were 10 times more likely to start pre-marital sexual debut than those watching one or less times a week [AOR = 10.15, 95% CI: (6.63,15.53)]. This finding is similar with studies in high school students of North East Ethiopia and Singapore [36,37]. Similarly, chewing khat is statistically associated with pre-marital sexual debut. Students who chewing khat were 9 times more likely to start pre-marital sexual debut compared to their counterparts [AOR = 8.99, 95% CI: (3.84, 21.06)]. This finding was supported by the studies in students of North East Ethiopia and in-school and out- of -school students in Ethiopia [16,37]. It may reason out as; most of the time after chewing khat, they drink alcohol [38]. Drinking alcohol decrease self control and predispose to risky behavior such as sexual intercourse [39-41].

Peer pressure was associated with pre-marital sexual debut. Youth who had peer pressure were three times more likely to initiate pre-marital sexual debut than their counterparts [AOR = 2.98, 95% CI = (1.57, 5.67)]. This finding was in line with other similar studies [9,11,32]; Possibly it might be; easier to discuss sensitive issues such as sexuality with peers than family members [42]. This discussion plays a significant role in influencing views, attitudes and sexual behavior of inexperienced youth to start pre-marital sexual debut [43].

### Strength of the study

High response rate, large sample size, sampling procedure and analysis methods utilized were appropriate to the study and considered as the strength of the study. The study provides useful information that will inform policy makers to design a strategy to increase number of unmarried female students who refrain from pre-marital sexual debut.

### Limitations of the study

This study was based on cross-sectional survey; causality cannot be inferred from our findings. Relies on self response for sensitive issues can invite social desirability bias and therefore underestimate prevalence of pre-marital sexual debut. The study was school based; therefore precludes generalization to all youths in Ethiopia indicating a need for further study using a more representative sample of youths in the country.

## Conclusion

In the study area significant number (30.8%) of unmarried high school female students practiced pre-marital sexual debut. Predominant factors associated with pre-marital sexual debut were peer pressure, age greater than 18 years, substance use like chewing khat and watching pornographic video.

### Recommendation

The finding suggests the need for communicating and supporting school students to help them make informed and safer decisions on their social and sexual behaviors. Therefore, Bahir dar city administration health and education bureau should design persistent and effective health education to decrease pre-marital sexual debut in unmarried female students.

## Competing interests

The authors declare that they have no competing interests.

## Authors’ contributions

YM: Conceived and designed the study, conducted statistical analysis and result interpretation, prepared manuscript. YB: assist in the design, statistical analysis, and interpretation of the result and reviewed the manuscript critically. Both authors read and approved the manuscript.

## Authors’ information

YM: BSC, MPH; I am working in Bahir Dar University, College of Medicine and Health Sciences, Bahir Dar, Ethiopia. YB: MD, MPH, PhD, Professor of Epidemiology and Public Health. Currently I am working in Addis Continental Institute of Public Health, Addis Ababa, Ethiopia.
